# Molecular genetic evidence of a novel morbillivirus in a long-finned pilot whale (Globicephalus melas).

**DOI:** 10.3201/eid0601.000107

**Published:** 2000

**Authors:** J K Taubenberger, M M Tsai, T J Atkin, T G Fanning, A E Krafft, R B Moeller, S E Kodsi, M G Mense, T P Lipscomb

**Affiliations:** Armed Forces Institute of Pathology, Washington, D.C., USA. taubenbe@afip.osd.mil

## Abstract

A long-finned pilot whale with morbilliviral disease was stranded in New Jersey. An immunohistochemical stain demonstrated morbilliviral antigen. Reverse transcriptase-polymerase chain reaction for morbillivirus P and N genes was positive. Novel sequences most closely related to, but distinct from, those of dolphin and porpoise morbilliviruses suggest that this virus may represent a third member of the cetacean morbillivirus group.


### Molecular Genetic Evidence of a Novel Morbillivirus in a Long-Finned Pilot Whale (*Globicephalus melas*)

**Jeffery K. Taubenberger,* Mark M. Tsai,* T. Joy Atkin,* Thomas G. Fanning,* Amy E. Krafft,* R.B. Moeller,* S.E. Kodsi,† M.G. Mense,† and Thomas P. Lipscomb***

*Armed Forces Institute of Pathology, Washington, D.C., USA; and
†Walter Reed Army Institute of Research, Washington, D.C., USA

A long-finned pilot whale with morbilliviral disease was stranded in New Jersey. An immunohistochemical stain demonstrated morbilliviral antigen. Reverse transcriptase-polymerase chain reaction for morbillivirus P and N genes was positive. Novel sequences most closely related to, but distinct from, those of dolphin and porpoise morbilliviruses suggest that this virus may represent a third member of the cetacean morbillivirus group.

In the last 12 years, newly recognized members of the morbillivirus family have caused many deaths among marine mammals, specifically cetaceans and pinnipeds (1). The first recognized marine mammal morbilliviral epizootic occurred in 1987-88 along the Atlantic coast of the United States (2,3). More than half the in-shore population of bottlenose dolphins (*Tursiops truncatus*) may have died. In 1988, thousands of harbor seals (*Phoca vitulina*) (4) and small numbers of harbor porpoises (*Phocoena phocoena*) (5) died in morbilliviral epizootics in northwestern Europe. A separate epizootic claimed thousands of striped dolphins (*Stenella coeruleoalba*) in the western Mediterranean (6). In 1993-94, another bottlenose dolphin epizootic occurred in the Gulf of Mexico (7,8). Recently, morbilliviral infection has been reported in cetaceans in the Pacific (9).

Viruses have been cultured from animals from some of the epizootics, and novel marine mammal morbilliviruses have been recognized. Two cetacean morbilliviruses have been identified and named porpoise morbillivirus (PMV) and dolphin morbillivirus (DMV). PMV was isolated from harbor porpoises that died along the Irish coast. DMV was first identified in striped dolphins from the Mediterranean (10). Since the characterization of these viruses, genetic identification has also been possible by reverse transcriptase-polymerase chain reaction (RT-PCR) and DNA sequence analysis in specimens from epizootics where virus was not cultured (11). These analyses have shown that both cetacean morbilliviruses (DMV and PMV) were present in the first recognized cetacean epizootic in bottlenose dolphins in 1987. While not species-specific in that study, only DMV was recovered in the striped dolphin epizootic in the Mediterranean, and only PMV sequences were noted from bottlenose dolphins in the subsequent Gulf of Mexico epizootic (11). Common dolphins (*Delphinus delphis*) stranded off the California coast from 1995 to 1997 were shown to have morbilliviral infection by virus neutralization and enzyme-linked immunosorbent assay (ELISA), as well as RT-PCR (9). Sequence analysis has shown that these animals were infected with PMV (Taubenberger et al., unpub. data).

We report a case of lethal morbilliviral infection in a long-finned pilot whale (*G. melas*). Sequence analysis of fragments of the morbillivirus phosphoprotein (P) and nucleoprotein (N) genes suggests that this is a novel morbillivirus, phylogenetically related to, but distinct from, the other cetacean morbilliviruses, PMV and DMV.

A female long-finned pilot whale (*G. melas*) was stranded below the deck of a beach home on the Delaware Bay, New Jersey. The whale was inactive and appeared near death. The likelihood

Address for correspondence: Jeffery K. Taubenberger, Armed Forces Institute of Pathology, Room G-137, 14th Street and Alaska Avenue, N.W., Washington, D.C. 20306-6000, USA; fax: 202-782-7623; e-mail: taubenbe@afip.osd.mil.

of recovery was remote, and euthanasia was considered appropriate. The animal was transported by truck to a veterinary diagnostic laboratory, where it died shortly after arrival.

At necropsy, body fat was found to be depleted. The lungs were dark red and congested. Nematodes were observed in the second stomach chamber. Significant histologic lesions were present in many organs. In the lung, alveoli and airways contained numerous neutrophils, histiocytes, and multinucleate cells, interspersed with cellular debris. Multifocal type 2 pneumocyte hyperplasia was observed. Many multinucleate cells and some bronchiolar epithelial cells contained eosinophilic intracytoplasmic and intranuclear inclusion bodies. An immunohistochemical stain demonstrated morbilliviral antigen (8) in bronchiolar epithelium, multinucleate (syncytial) cells, and type 2 pneumocytes (Figure 1). Syncytial cells, often containing eosinophilic intracytoplasmic and intranuclear inclusion bodies, were also observed in spleen, lymph nodes, and liver. Additionally, eosinophilic intracytoplasmic inclusion bodies were present in colonic and gastric epithelia and in transitional epithelium of the kidney. Intracytoplasmic and intranuclear inclusion bodies were seen in glossal epithelium. Other histologic lesions included nonsuppurative encephalomyelitis, necrotizing hepatitis, erosive colitis, necrotizing gastritis, ulcerative glossitis, and ulcerative esophagitis.

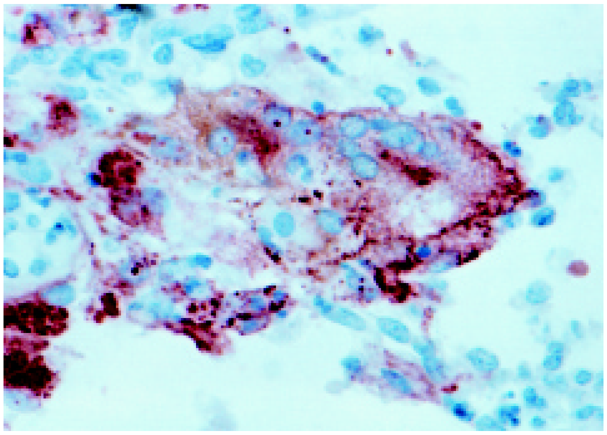

Figure 1. Lung of pilot whale. Positive intracytoplasmic and intranuclear immunoperoxidase staining of morbilliviral antigen in syncytial cell (center) and bronchiolar epithelium (lower left). Avidin-biotin-peroxidase with Harris' hematoxylin counterstain. Original magnification 594x.

RT-PCR was performed on RNA isolated from formalin-fixed, paraffin-embedded lung and brain tissues (7,11). A 378 nucleotide fragment of the P gene (GenBank accession number AF200817 and a 230 nucleotide fragment of the N gene (GenBank accession number AF200818 were amplified and sequenced. In both cases the sequences were novel but related to the previously described cetacean morbillivirus sequences. We have tentatively named this virus "pilot whale morbillivirus" or PWMV.

Sequences of both the N and P gene fragments were analyzed by parsimony and neighbor-joining (Figure 2). Parsimony analyses (12) with Sendai virus as outgroup placed PWMV in the cetacean morbillivirus clade with PMV and DMV, with moderate-to-high bootstrap values (N sequence = 70; P sequence = 100). Neighbor-joining (13) also placed PWMV in the cetacean morbillivirus clade with high bootstrap values (N sequence = 97; P sequence = 100). Thus PWMV is clearly related to, but distinct from, PMV and DMV, and the latter two are more closely related to one another than either is to PWMV (Figure 2).

Both the origin of these cetacean morbilliviruses and the mechanism of spread to different parts of the world remain unknown. Recently, serologic evidence of morbilliviral infection was obtained from 11 of 15 species of odontocete cetaceans of the western Atlantic (14), suggesting that exposure to these viruses has been widespread. Serologic evidence of enzootic infection (with a seroprevalence of 86%) was also obtained from two species of pilot whales (*G. melas* and *G. macrorhynchus*) in the western Atlantic (15). Clinical disease consistent with morbilliviral pneumonia was detected in one *G. melas* calf, although the morbillivirus in that case was not characterized. Duignan et al. (15) speculated that pilot whales may serve as vectors of morbillivirus infection to other odontocete cetaceans.

The results from this case suggest that pilot whales may have their own species-adapted morbillivirus. Given that this is a single case, that no morbilliviral epizootic has been described for pilot whale species, and that seroprevalence is consistent with enzootic morbilliviral infection (15), lethal infection might be rare in these species. Nevertheless, it remains possible for pilot whales to serve as vectors by which

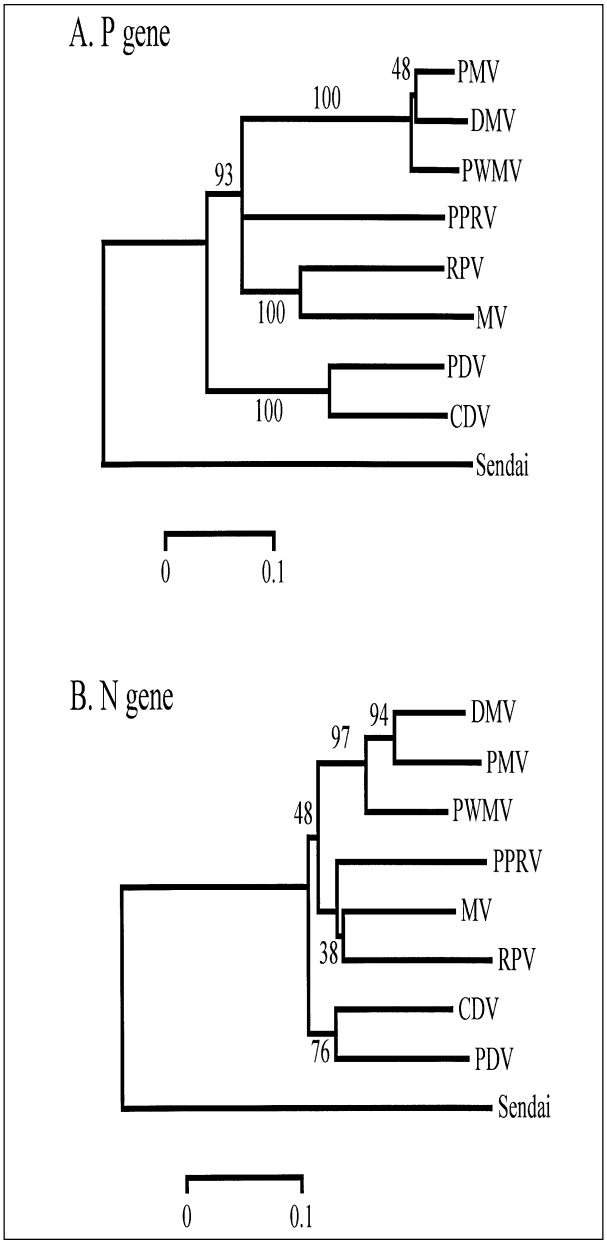

Figure 2. Neighbor-joining analyses of partial P and N gene sequences with branch distances as shown. Analyses were performed with MEGA, version 1.01 (13). For the P gene, a 378 nucleotide fragment was amplified (7,8) using the following primers: 5'-CGGAG ACCGAGTCT-TCATT-3' (forward) and 5'-ATTGGGTTGC ACCACT-TG TC-3' (reverse), corresponding to nucleotides 2190 to 2567 as aligned to the measles virus P gene (Edmonston strain). For the N gene, a 230-nucleotide fragment was amplified using the following primers: 5'-CCHAGRATYGCTGAAATGATHTGTGA-3' (forward) and 5'-AACTTG TTCTGRATWGAGTTYTC-3' (reverse), corresponding to nucleotides 849 to 1078, as aligned to the measles virus N gene (Edmonston strain, GenBank accession number Z66517). RT-PCR and sequence analysis were performed as described (7,8).

immunologically naïve species are exposed to morbillivirus infection. The viruses in these hosts may have undergone species-adaptive changes, reflecting the genetic differences between DMV, PMV, and PWMV. While only partial sequences have been obtained from two of the morbilliviral genes in this case, the fact that it is closer to the root of the cetacean morbillivirus clade (Figure 2) suggests that PWMV resembles the common ancestor of the clade more closely than either DMV or PMV.

### Acknowledgments

We thank the Marine Mammal Stranding Center, Brigantine, New Jersey, for collecting and transporting the whale; Sionagh H. Smith for performing the necropsy; and R.-A. V. Ferris for assistance with the photomicrograph.

This study was partially funded by the National Marine Fisheries Service.

Dr. Taubenberger is chief of the division of Molecular Pathology, department of Cellular Pathology, Armed Forces Institute of Pathology, Washington, D.C., U.S.A. His areas of expertise include molecular pathology, RNA viruses, and immunology. His research interests include genetic characterization of the 1918 influenza virus and marine mammal morbilliviruses, genetic changes in breast cancer and lymphomas, and the development of B and T lymphocytes.

### References

1. Kennedy S. Morbillivirus infections in aquatic mammals. J Comp Pathol 1998;119:201-25.
2. Lipscomb TP, Schulman FY, Moffett D, Kennedy S. Morbilliviral disease in Atlantic bottlenose dolphins (*Tursiops truncatus*) from the 1987-1988 epizootic. J Wildl Dis 1994;30:567-71.
3. Schulman FY, Lipscomb TP, Moffett D, Krafft AE, Lichy JH, Tsai MM, et al. Reevaluation of the 1987-88 Atlantic coast bottlenose dolphin (*Tursiops truncatus*) mortality event with histologic, immunohistochemical, and molecular evidence for a morbilliviral etiology. Vet Pathol 1997;34:288-95.
4. Osterhaus ADME, Groen J, Spijkers HEM, Broeders HWJ, UytdeHaag FGCM, de Vries P, et al. Mass mortality in seals caused by a newly discovered morbillivirus. Vet Microbiol 1990;23:343-50.
5. Kennedy S, Smyth JA, Cush PF, McAliskey M, McCullough SJ, Rima BK. Seven histopathologic and immunocytochemical studies of distemper in harbor porpoises. Vet Pathol 1991;28:1-7.
6. Domingo M, Vilafranca M, Visa J, Prats N, Trudgett AL, Visser I. Evidence of chronic morbillivirus infection in the Mediterranean striped dolphin (*Stenella coeruleoalba*). Vet Microbiol 1995;44:229-39.

7. Krafft AE, Lichy JH, Lipscomb TP, Klaunberg BA, Kennedy S, Taubenberger JK. Postmortem diagnosis of morbillivirus infection in bottlenose dolphins (*Tursiops truncatus*) in the Atlantic and Gulf of Mexico epizootics by a polymerase chain reaction-based assay. J Wildl Dis 1995;31:410-5.
8. Lipscomb TP, Kennedy S, Moffett D, Krafft AE, Klaunberg BA, Lichy JH, et al. Morbilliviral epizootic in Atlantic bottlenose dolphins of the Gulf of Mexico. J Vet Diagn Invest 1996;8:283-90.
9. Reidarson TH, McBain J, House C, King DP, Stott JL, Krafft AE, et al. Morbillivirus infection in stranded common dolphins (*Delphinus delphis*) from the Pacific Ocean. J Wildl Dis 1998;34:771-6.
10. Barrett T, Visser IKG, Mamaev L, Goatley L, van Bressem M-F, Osterhaus ADME. Dolphin and porpoise morbilliviruses are genetically distinct from phocine distemper virus. Virology 1993;193:1010-2.
11. Taubenberger JK, Tsai MM, Krafft AE, Lichy JH, Reid AH, Schulman FY, et al. Two different morbilliviruses implicated in bottlenose dolphin epizootics. Emerg Infect Dis 1996;2:213-6.
12. Swofford DL. PAUP: Phylogenetic Analysis Using Parsimony, version 3.1.1 (Illinois Natural History Survey, Champaign, Illinois; 1991.
13. Kumar S, Tamura K, Nei M. Molecular evolutionary genetics analysis, version 1.01. Pennsylvania State University, University Park, Pennsylvania; 1993.
14. Duignan PJ, House C, Geraci JR, Duffy N, Rima BK, Walsh MT, et al. Morbillivirus infection in cetaceans of the western Atlantic. Vet Microbiol 1995;44:241-9.
15. Duignan PJ, House C, Geraci JR, Early G, Copland HG, Walsh MT, et al. Morbillivirus infection in two species of pilot whales (*Globicephala* sp.) from the western Atlantic. Marine Mammal Science 1995;11:150-62.
